# Health systems thinking: A new generation of research to improve healthcare quality

**DOI:** 10.1371/journal.pmed.1002682

**Published:** 2018-10-30

**Authors:** Hannah H. Leslie, Lisa R. Hirschhorn, Tanya Marchant, Svetlana V. Doubova, Oye Gureje, Margaret E. Kruk

**Affiliations:** 1 Department of Global Health and Population, Harvard T. H. Chan School of Public Health, Boston, Massachusetts, United States of America; 2 Departments of Medical Social Sciences and Psychiatry and Behavioral Sciences, Northwestern University Feinberg School of Medicine, Chicago, Illinois, United States of America; 3 Department of Disease Control, Faculty of Infectious and Tropical Diseases, London School of Hygiene & Tropical Medicine, London, United Kingdom; 4 Epidemiology and Health Services Research Unit, Instituto Mexicano del Seguro Social, Mexico City, Mexico; 5 WHO Collaborating Centre for Research and Training in Mental Health, Neuroscience, Drug and Alcohol Abuse, University of Ibadan, Ibadan, Nigeria

## Abstract

Hannah Leslie and colleagues of the High-Quality Health Commission discuss in an Editorial the findings from their report that detail the improvements needed to prevent declines in individuals’ health as the scope and quality of health systems increase. Patient-centered care at the population level, improved utility of research products, and innovative reporting tools to help guide the development of new methods are key to improved global healthcare.

As global and national pursuit of universal health coverage (UHC) accelerates, health system quality has emerged as a critical concern, a weakness that could blunt the promise of UHC. Without adequate quality of care, access to care and financial protection will be insufficient to improve population health. Findings from the recent Lancet Global Health Commission for High Quality Health Systems suggest that increasing the scope and reach of health systems without attention to improving quality has the potential to worsen health [1]: individuals can be harmed by unsafe procedures or unnecessary treatment, communities may lose faith in health systems, and already limited resources will be misdirected. Health systems in low- and middle-income countries (LMICs) today demonstrate broad and deep gaps in readiness to provide care [2–5], systematic deficiencies in diagnosis and treatment [6–8], and frequent reports of disrespectful care [9]. As of 2016, receipt of poor-quality care resulted in an estimated 5 million excess deaths in LMICs [10]. The breadth of this quality deficit—spanning long-standing global health priorities such as maternal [11] and child health [4], as well as understudied needs such as mental healthcare [12]—demands a new generation of research to compel change.

Central to a productive new research agenda is the recognition of health system quality as a critical factor in progress towards UHC. Research scope and methodology must advance to match the magnitude and urgency of this challenge. Health systems are complex adaptive systems with multiple interconnected levels and overlapping sectors [13,14]. Individuals across diverse populations are active agents in choosing if, when, and where to access care [15]. Amidst these complexities, decision-makers need research that addresses national priorities and reflects the complexity and time frame for impact. Current research is inadequate to meet this need.

We suggest three core characteristics for ensuring that research delivers the actionable knowledge required to achieve UHC: meaning, utility, and innovation.

## Meaning: Answer questions that matter for population health

Conducting meaningful research depends upon a common language and valid measures to benchmark progress and inform action. A peril of the current global attention to health system quality is the elasticity of central concepts concerning quality care and people-centered health systems. Without shared understanding and standards among researchers and policy makers, momentum is in danger of dissipating into inconsistent assessments and mutually incomparable investments, leaving researchers without insight and policy makers without direction.

Health system quality encompasses much more than inputs like staffing, equipment, and drugs. Such indicators of facility infrastructure provide limited insight on quality of care received [16]. Further, assessments at a single point in time will not capture the dynamics of system–population interactions that shape health outcomes [17]. To replace crude indicators such as childbirth in a health facility, assessment must not stop at the step of asking how many women delivered at a facility that might be capable of providing appropriate care but must fundamentally shift towards determining whether women were treated with respect, had complications identified quickly and managed appropriately, and returned home with a healthy newborn and the confidence to seek care for herself and her child in the future.

Patient centeredness is a key attribute of high-quality health systems [18] but is at risk of becoming a platitude unless research can operationalize this idea within a context-specific quality framework. Patient ratings of health services are a function not just of quality of care but of individuals’ own expectations and capacity for self-advocacy, which are fundamentally patterned by inequities. Those most frequently expressing satisfaction are likely to be the most vulnerable to poor quality [1]. Judging a health system or improvement strategy based only on satisfaction risks worsening existing inequities. To capture more than low expectations, tools for efficient assessment of patient experience and trust in the health system must be validated and used to assess strategies for patient-centered care.

## Utility: Align research products with user needs

The dominance of global rather than national funders for health system research and the privileging of short-term, program-specific effects over breadth and sustainability has resulted in fragmented assessments that are poorly suited to sound policy making [1,16]. Country-level action is needed to guide the next generation of research towards system-wide improvement. Strengthening quality measurement and implementing large-scale improvements demands a capacity for synthesizing complex data that goes beyond current mechanisms for collection and analysis. Countries must strengthen capacity to extract meaningful insight about system quality from diverse data sources—demographics, health information systems, finance, and patient sources, among others—to inform decisions across health system levels.

Research should identify cross-cutting measures that can serve as proxies for health system quality and its impacts. Such efforts must be systems oriented, seeking evidence that accounts for unintended outcomes—positive or negative—across service areas and levels of care. For instance, provision of quality mental healthcare depends on a system capable of screening and detection, integration, and continuous care [19]. How should development of indicators, analytic tools, and data-use plans to measure quality of mental healthcare differ if the goal is to provide insight on system functioning as a whole rather than on a single condition or capacity? Given the resource and time costs of measurement, any new measures should be assessed not just on validity but based on their feasibility, intended purpose, and specific contribution to improvement at a system level. Established measures of lower utility should be phased out. Evaluations should also provide the information required to build political will for large-scale change: a focus on short-term effects at the point of care is unlikely to do so. Quality assessment that captures only inputs, that compares patient health outcomes without accounting for population risk and individual health status, or that considers patient satisfaction apart from vulnerabilities and expectations will yield little gain.

## Innovation: Develop new methods to measure and evaluate

In measurement, innovation is needed in tools and methods to collect data on patient experience and outcomes, to extract insight on quality from health facilities and systems, and particularly to synthesize information for practical application. In improvement, tools are needed for broad-scale evaluation that balances feasibility against rigorous assessment of causality and transferability. Pooling insight and tools across traditional disciplinary divides and looking beyond health science to education and organizational management can accelerate progress. Solutions must consider representativeness as well as vulnerable subpopulations to ensure this progress is equitable.

For health systems to contribute to improved population health, innovative approaches must change the trajectory of research to situate questions within complex systems and extract insights beyond the handful of currently studied health needs and individual clinics. To gain insight over time and across conditions and settings will require a shift towards coordinated, country-led efforts. These in turn demand coordination among researchers across disciplines and settings to extract and translate valuable ideas and greater flexibility in resource allocation from national and global funders to accommodate the complexity of research producing generalizable insights.

The unprecedented attention to quality of care in 2018 [20] has amply demonstrated the magnitude of the challenge facing national governments, multilateral organizations, and researchers. The research questions and methods prioritized now will determine whether the momentum from these efforts translates over the next decade into the insights needed to build better health systems.

## Abbreviations

LMIC: low- and middle-income country
UHC: universal health coverage
